# Immune-related redox metabolism of embryonic cells of the tick *Rhipicephalus microplus* (BME26) in response to infection with *Anaplasma marginale*

**DOI:** 10.1186/s13071-017-2575-9

**Published:** 2017-12-19

**Authors:** Sandra Patricia Kalil, Rafael Diego da Rosa, Janaína Capelli-Peixoto, Paula Cristiane Pohl, Pedro Lagerblad de Oliveira, Andrea Cristina Fogaça, Sirlei Daffre

**Affiliations:** 1 Department of Parasitology, Institute of Biomedical Sciences, University of São Paulo, São Paulo, São Paulo 05508-900 Brazil; 2 Laboratory of Biochemistry of Hematophagous Arthropods, Institute of Medical Biochemistry Leopoldo de Meis, Federal University of Rio de Janeiro, Rio de Janeiro, Rio de Janeiro 21941-909 Brazil; 3 Present address: Laboratory of Immunology Applied to Aquaculture, Department of Cell Biology, Embryology, and Genetics, Federal University of Santa Catarina, Florianópolis, Santa Catarina 88040-900 Brazil

**Keywords:** Anaplasmosis, Redox metabolism, Rickettsiae, Tick immunity, ROS

## Abstract

**Background:**

It is well known that reactive oxygen species (ROS) and reactive nitrogen species (RNS) are involved in the control of pathogens and microbiota in insects. However, the knowledge of the role of ROS and RNS in tick-pathogen and tick-microbiota interactions is limited. Here, we evaluated the immune-related redox metabolism of the embryonic cell line BME26 from the cattle tick *Rhipicephalus microplus* in response to *Anaplasma marginale* infection.

**Methods:**

A high-throughput qPCR approach was used to determine the expression profile of 16 genes encoding proteins involved in either production or detoxification of ROS and RNS in response to different microbial challenges. In addition, the effect of RNAi-mediated gene silencing of catalase, glutathione peroxidase, thioredoxin and protein oxidation resistance 1 in the control of infection with *A. marginale* was evaluated.

**Results:**

Infection with *A. marginale* resulted in downregulation of the genes encoding ROS-generating enzymes dual oxidase and endoplasmic reticulum oxidase. In contrast, the genes encoding the antioxidant enzymes superoxide dismutase, catalase, glutathione peroxidase, glutathione S-transferase, thioredoxin, thioredoxin reductase and peroxiredoxin were upregulated. The gene expression pattern in response to infection with *Rickettsia rickettsii* and exposure to heat-killed microorganisms, *Micrococcus luteus*, *Enterobacter cloacae* or *S. cerevisiae* was the opposite of that triggered by *A. marginale* challenge. The simultaneous silencing of three genes, catalase, glutathione peroxidase, and thioredoxin as well as the oxidation resistance 1 gene by RNAi apparently favoured the colonization of BME26 cells by *A. marginale*, suggesting that the antioxidant response might play a role in the control of infection.

**Conclusions:**

Taken together, our results suggest that a general response of tick cells upon microbial stimuli is to increase ROS/RNS production. In contrast, *A. marginale* infection triggers an opposite profile, suggesting that this pathogen might manipulate the tick redox metabolism to evade the deleterious effect of the oxidant-based innate immune response.

**Electronic supplementary material:**

The online version of this article (10.1186/s13071-017-2575-9) contains supplementary material, which is available to authorized users.

## Background

Reactive oxygen species (ROS) are highly reactive oxygen-derived molecules with the potential to damage key cellular components, including lipids, proteins, and DNA. ROS also have an essential role in infection-related physiological and pathophysiological processes such as signalling, control of tissue injury and inflammation, as well as cell survival, proliferation, differentiation, and apoptosis [1, 2]. In the immune response to pathogens, ROS and reactive nitrogen species (RNS) are rapidly generated by specific enzymes in many cell types. These enzymes include NADPH oxidase, which produces superoxide anion (O_2_^−^); dual oxidase (DUOX) and endoplasmic reticulum oxidoreductin (ERO1), which produce hydrogen peroxide (H_2_O_2_); and nitric oxide synthase (NOS), which generates nitric oxide (NO) [3–5]. Cells have developed strategies to counteract the potential damage of ROS via detoxification of these molecules and repair of damage induced by ROS. In this respect, superoxide dismutase (SOD) converts O_2_^−^ into H_2_O_2_ whereas catalase (CAT) and glutathione peroxidase (GPX) detoxify H_2_O_2_ into H_2_O and O_2_ [6]. Thioredoxins (TRX), which are a partner of reduced/oxidized glutathione in redox regulation, catalyze the reversible reduction of protein disulphide bonds, while the cysteine residues of TRX active-site are reduced by both TRX reductase (TRXR) and NADPH [7]. Peroxiredoxins (PRX) are a group of non-seleno thiol-specific peroxidases that contribute to cellular redox control by eliminating organic hydroperoxides and H_2_O_2_. In addition, phospholipid hydroperoxide glutathione peroxidases (PHGPX) catalyze the reduction of H_2_O_2_ using glutathione as an electron donor [8]. Finally, glutathione S-transferases (GST) catalyze the conjugation of GSH and electrophilic compounds, increasing the solubility of products and facilitating their excretion [9].

In arthropods, the triggering of ROS/RNS production is a key component of the response to pathogens [10–13]. In the midgut of *Drosophila*, DUOX is essential to control the number of pathogenic microorganisms [10]. In the midgut of *Anopheles gambiae*, infection with *Plasmodium berghei* decreased H_2_O_2_ detoxification, which limits parasite survival, suggesting that ROS is involved in modulating mosquito immunity [13]. The same research group showed that silencing the gene encoding the protein oxidation resistance 1 (OXR1) increased the systemic levels of H_2_O_2_ and consequently decreased *P. berghei* infection [11]. In mosquitoes, it has also been shown that DUOX, together with a heme-peroxidase, promotes the formation of a dityrosine bond between extracellular proteins, forming a network that prevents immune activation by the gut microbiota [12].

The process of redox-based innate immune effectors in response to pathogen infection is much less understood in ticks. Our research group demonstrated that O_2_^−^ and H_2_O_2_ were produced by the hemocytes of the cattle tick *Rhipicephalus microplus* in response to a microbial challenge with *Micrococcus luteus*, zymosan, and phorbol 12-miristate 13-acetate (PMA) [14]. Moreover, analogous to what had been described for the mosquitoes, Yang et al. [15] suggested that DUOX/peroxidase system is important for the survival of *Borrelia burgdorferi* in the gut of the tick *Ixodes scapularis*. Similar to the results observed for mosquitoes [12], the silencing of either DUOX or peroxidase impaired the acellular gut barrier formed by tyrosine cross-linking (dityrosine) and thus reduced the load of *B. burgdorferi* in the tick gut. Importantly, the induction of *NOS* gene expression and activity by disruption of the dityrosine network promoted a decrease of bacterial load [15].

Our research group is interested in understanding the immune response of *R. microplus* during infection with *Anaplasma marginale*, the etiological agent of bovine anaplasmosis*.* This disease causes significant economic losses due to temporary infertility, abortion, increased mortality, and high costs of treatment [16]. We have previously reported significant differences in the transcriptional expression profile of genes encoding components of tick immune signaling pathways (Toll, IMD, JNK, and Jak-Stat) in non-infected BME26 cells (derived from *R. microplus* embryos) in comparison to cells harboring either *A. marginale* or *Rickettsia rickettsii*, the causative agent of Rocky Mountain spotted fever [17]. Most of the analyzed genes were downregulated by *A. marginale* infection, suggesting that this pathogen might manipulate the tick immune system, favouring bacterial survival and colonization. In contrast, the expression of most of the genes from immune signalling pathways in *R. rickettsii*-infected cells was upregulated. Moreover, another study of our group revealed that the IMD signalling pathway controls *A. marginale* infection in adult male *R. microplus* ticks [18].

Here, we assessed the role of immune-related redox metabolism in the control of *A. marginale* infection in BME26 cells. First, we determined the differential expression profile of redox metabolism genes in BME26 cells exposed to microbial stimuli, including two alive pathogens naturally transmitted by ticks, *A. marginale* and *R. rickettsii*, and three heat-killed microorganisms not transmitted by ticks (*M. luteus*, *Enterobacter cloacae* and *Saccharomyces cerevisiae*). Differently, from other microbial stimuli, *A. marginale* infection upregulated the majority of antioxidant genes while most of the pro-oxidant genes were downregulated. In addition, the silencing of the genes encoding proteins involved in ROS detoxification, catalase, glutathione peroxidase, thioredoxin and oxidation resistance 1 by RNAi decreased the load of *A. marginale* in BME26 cells. These results suggest that *A. marginale* might manipulate the tick redox mechanism favouring its survive. However, it cannot be ruled out that host cell response controls infection.

## Methods

### Tick cell lines and microorganisms

The embryonic cell lines BME26, derived from *R. microplus* [19], and ISE6, derived from *I. scapularis* [20], were cultured as previously described [19]. Cell growth and viability were assessed by cell counting in a Neubauer chamber using optical microscopy after trypan blue staining. The microorganisms used in the experiments were the Gram-positive bacterium *Micrococcus luteus* (ATCC 9341A), the Gram-negative bacterium *Enterobacter cloacae* K12 (provided by Dr Hans G. Boman, Stockholm University, Sweden), the yeast *Saccharomyces cerevisiae* (ATCC 208353) and the rickettsiae *Anaplasma marginale* (Jaboticabal strain) [21] and *Rickettsia rickettsii* (Taiaçu strain) [22].

### Nucleic acid extraction and cDNA synthesis

Total RNA and genomic DNA (gDNA) were extracted from BME26 cells using TRIzol® reagent (Thermo Fisher Scientific, Waltham, USA) and Smarter Nucleic Acid Sample Preparation (STRATEC Molecular, Berlin, Germany), respectively, as described previously [17]. RNA samples were treated with DNase I (Thermo Fisher Scientific) to eliminate contaminant gDNA, and cDNA was synthesized using 250–1000 ng of purified total RNA as template in a 20 μl reaction volume containing 2.5 μM oligo(dT), 0.5 mM dNTPs, 5 mM DTT, 2 units of RNaseOUT ribonuclease inhibitor (Thermo Fisher Scientific), and 10 units of SuperScript III reverse transcriptase (Thermo Fisher Scientific), according to the manufacturer’s instruction. As a control, an aliquot of treated RNA was used in PCR reactions to confirm the elimination of residual genomic DNA (data not shown).

### Quantification of *A. marginale* and *R. rickettsii* by real-time quantitative PCR

The number of rickettsiae in BME26 cells was determined by quantitative real-time PCR (qPCR) using specific primers and a hydrolysis probe for the genes that encode the major surface protein 5 (*msp5*) of *A. marginale* [23] and citrate synthase of *R. rickettsii* (*gltA*) [24]. Briefly, the amplification reaction was performed in three technical replicates in a final volume of 15 μl containing 2 μl of gDNA (approximately 100 ng), 2 μl of a mixture of forward and reverse primers (0.6 μM each), 0.02 μl of *Taq*Man probe, 7.5 μl of Maxima Probe/ROX qPCR Master Mix (Thermo Fisher Scientific), and 3.5 μl of ultrapure water. The reactions were performed on a StepOnePlus™ Real-Time PCR System (Thermo Fisher Scientific) using the following thermocycler programs: 10 min at 95 °C, followed by 40 cycles of 15 s at 95 °C, 30 s at 60 °C, and 45 s at 72 °C *(A. marginale)* and 10 min at 95 °C, followed by 40 cycles of 15 s at 95 °C, 15 s at 55 °C, and 30 s at 72 °C (*R. rickettsii*). In each analysis, the cycle of quantification (C_q_) values of reactions using a dilution series of 10^2^ to 10^7^ copies of a plasmid (pGEM-®T Easy, Promega, Madison, USA) containing a fragment of either *msp5* or *gltA* were used to establish a standard curve for determining the absolute number of *A. marginale* and *R. rickettsii*, respectively. To that end, those fragments were previously sequenced to confirm their identity to respective sequences in public databases.

### Primers

The primer design for the high-throughput microfluidic RT-qPCR assay was made manually, and the parameters included a primer annealing temperature of 58 °C to 62 °C, primer length of 20 to 21 bp, primer G/C content of approximately 50%, and amplicon length of 100–200 bp. Potential self-complementarity and primer-dimer formation were checked using FastPCR Professional software (http://primerdigital.com/fastpcr.html). The efficiency of primers used in RT-qPCR was calculated in the BioMark Real-Time PCR System, and only primers with an amplification efficiency above 90 (linear C_q_ values) were accepted. All primers used in this study are fully detailed in Additional file 1.

### High-throughput microfluidic RT-qPCR

The microbial stimuli of BME26 cells, extraction of nucleic acids, high-throughput microfluidic RT-qPCR, and data analysis were performed simultaneously to the previously study of our research group [17]. As recognized by the Minimum Information for Publication of Quantitative Real-Time PCR Experiments, the MIQE Guidelines [25], the expression of different candidate genes for RT-qPCR data normalization was examined. In our analysis, eight candidate genes were considered: elongation factor-1 alpha (*ef-1α*), glyceraldehyde-3-phosphate dehydrogenase (*gapdh*), beta-actin (*actb*), malate dehydrogenase (*mdh*), glutamate dehydrogenase (*gdh*), cytochrome *c* oxidoreductase (*cypor*), 40S ribosomal protein S3A (*RIBS3A*) and ribosomal protein L4 (*rpl4*). Their expression levels were analyzed individually (C_q_ values) and in combination (geometric mean of C_q_ values) [26]. For our experimental conditions, the best reference for qPCR normalization in BME26 cells was the geometric mean of the C_q_ values of *mdh*, *gdh*, *cypor* and *40SRPS3A* regarding both C_q_ variation and primer efficiency.

### RNAi gene-silencing assay

To verify the importance of an antioxidant response of BME26 cells on *A. marginale* infection, the encoding genes of catalase (GenBank: CV451339), *gpx* (GenBank: CV440147), *trxr* (GenBank: CV451339) and *oxr*1 (GenBank: MF503617) were silenced using RNA-mediated interference (RNAi). The encoding gene of the merozoite surface protein 1 (*msp*1) of *Plasmodium falciparum* was used as control (GenBank: AF061132). Target DNA fragments were amplified using cDNA from BME26 cells or a plasmid containing *msp*1 and primers designed for *Rmcat*, *Rmgpx*, *Rmtrxr*, *Rm oxr*1, or *Pfmsp*1 containing the T7 polymerase promoter sequence (Additional file 1: Table S1). The PCR cycling conditions were: 95 °C for 30 s followed by 30 cycles of 55 °C for 1 min and 72 °C for 1 min. PCR products were analyzed by agarose gel electrophoresis and purified using the GeneJet Purification kit (Fermentas, Vilnius, Lithuania). dsRNAs were synthesized using the T7 RiboMax™ Express RNAi System kit (Promega) and 950 ng of PCR-amplified DNA fragments as template. After dsRNA synthesis, the specificity was checked by electrophoresis in agar gel. Only dsRNA with a unique band with the expected size were used.

BME26 cells (1.5 × 10^6^) were sub-cultured into flasks for 24 h before the addition of 10^13^ dsRNA molecules in L15B infection medium [27]. The BME26 cells were incubated simultaneously with a mixture of ds*cat*, ds*gpx*, and ds*trxr*, or ds*oxr*1 alone; ds*msp*1 was used as a control. After 24 h, BME26 cells were challenged with *A. marginale*. To that end, frozen cultures of *A. marginale* in ISE6 cells were used (3 × 10^8^ bacteria/vial) were thoroughly lysed by subjecting the samples to three cycles of freezing in liquid nitrogen followed by thawing in a water-bath at 37 °C. Then, the suspension was centrifuged at 500× *g* for 5 min at 4 °C, and the same volume of supernatant was added as inoculum in all experiments. An aliquot of the inoculum was stored for quantification of bacteria along with the aliquots collected from each time point. The quantification of the inoculum was further used to calculate the rickettsiae infection ratio of each experiment. Therefore, ratios may vary among assays from 1:9 to 1:90. After incubation for additional 72 h, the supernatant was discarded, the cell monolayer was washed with PBS and then detached with a scraper or Trypsin-EDTA solution (Vitrocell, Campinas, Brazil). The cell suspension was centrifuged at 3000× *g* for 10 min at 4 °C. RNA and gDNA were extracted from resulting pellet and used for evaluation of gene silencing and bacterial quantification, respectively. The expression of target genes (*cat*, *gpx, trxr, oxr*1) and the endogenous reference gene (40S ribosomal protein *S3a*) was assessed by SYBR green quantitative real-time PCR (qPCR). The reactions were performed on a StepOnePlus™ Real-Time PCR System (Thermo Fisher Scientific). The PCR condition was 95 °C for 10 min followed by 40 cycles of 95 °C for 15 s and 60 °C for 1 min. Gene expression was analyzed as described for the high-throughput microfluidic RT-qPCR experiment [17]. The number of *A. marginale* in BME26 cells was determined by qPCR. Five and eight biological replicates (flasks of BME26 cells) were processed for each group (treated with either control or target gene dsRNA) in the triple and *oxr*1 silencing experiments, respectively. The infection ratio was 1:9 (triple silencing) and 1:64 (*oxr*1 silencing).

### Quantitation of H_2_O_2_

H_2_O_2_ was measured with Amplex Red (Invitrogen) following the recommendations of the manufacturer (Amplex® Red Hydrogen Peroxide/Peroxidase Assay Kit, Thermo Fisher Scientific). Before the bacterial challenge, 5 × 10^4^ BME26 cells were sub-cultured in 24-well plates for 24 h followed by two washes with Krebs-Ringer phosphate buffer with glucose (KRPG) (145 mM NaCl, 5.7 mM sodium phosphate, 4.86 mM KCl, 0.54 mM CaCl_2_, 1.22 mM MgSO_4_, 5.5 mM glucose, pH 7.35) and incubation in KRPG containing 100 μM Amplex Red (10-acetyl-3,7-dihydroxyphenoxazine) and 0.2 U/ml horseradish peroxidase (HRP). Tick cells were challenged with *A. marginale* (ratio 1:34) obtained as described above (RNAi gene-silencing assay). Uninfected ISE6 cell lysate prepared as described for *A. marginale* inoculum was used as a control. In addition, BME26 cells incubated with 125 μM menadione served as the positive control [28, 29]. At 1, 6, 24 and 72 h post-challenge at 34 °C in the dark, the incubation medium was centrifuged for 2 min at 10000× *g*, and the supernatant was transferred to cell culture plates (Corning Costar**®;** CLS3991, Sigma-Aldrich). The fluorescence was measured using a SpectraMax 190 (Molecular Devices, Sunnyvale, USA) with excitation at 560 nm and emission at 590 nm and was compared to a standard curve containing 0.05 to 500 pmol of H_2_O_2_. Three biological samples were analyzed for each treatment.

### Statistical analysis

The differences between treatments were analyzed statistically by Mann-Whitney test using GraphPad Prism version 7.0 for Windows (GraphPad Software, San Diego, CA, USA). A *P*-value lower than 0.05 was considered statistically significant.

## Results

### Redox balance genes are differentially expressed in BME26 cells upon microbial stimuli

First, we evaluated *A. marginale* and *R. rickettsii* growth in BME26 cells and the cell viability along infection (Table 1). The infection ratios with rickettsiae were based on the kinetics of growth previously standardized by our research group. As *R. rickettsii* grows faster than *A. marginale* and it is quite more virulent, causing the death of the tick cells, we used an inoculum approximately 10^4^ lower of *R. rickettsii* than of *A. marginale*. The total number of *A. marginale* remained relatively constant (average of 5.57 × 10^7^ rickettsiae/flask) over the 72 h infection period, and the mortality rate of BME26 cells was low (less than 20%). In contrast, the average number of *R. rickettsii* increased during infection and reached 1.61 × 10^6^ bacteria/flask over 48–72 h post-infection. *R. rickettsii* counts in BME26 cells were below the detection threshold of the absolute quantification by qPCR at 6 and 24 h (Table 1). Although no significant differences in the number of *R. rickettsii* were found between 48 and 72 h, the mortality of BME26 cells reached approximately 45% at 72 h post-infection. It is important to note that at 48 h and 72 h post-infection, the quantity of the two rickettsiae in BME26 cells differs by only one order of magnitude (Table 1).

**Table 1:** Rickettsial quantification in BME26 cells

Post-infection (h)	*A. marginate* infection		*R. rickettsii* infection	
	Number of bacteria/flask	% BME26 mortality	Number of bacteria/flask	% BME26 mortality
6	4.21 × 10^7^ ± 1.15 × 10^7^	10 ± 2	nd	4 ± 0.8
24	6.66 × 10^7^ ± 6.39 ± 10^6^	16 ± 4	nd	11 ± 2
48	4.86 × 10^7^ ± 7.77 × 10^6^	16 ± 8	2.04 × 10^6^ ± 4.32 × 10^5^	7 ± 3
72	6.55 × 10^7^ ± 2.38 × 10^7^	19 ± 6	1.18 × 10^6^ ± 4.52 × 10^5^	45 ± 4

The infection of BME26 cells was monitored by absolute qPCR. The mortality of BME26 cells was monitored by trypan blue staining under light microscopy (adapted from Rosa et al. [17]). *Abbreviation*: *nd*, not detected by qPCR

**Fig. 1:**
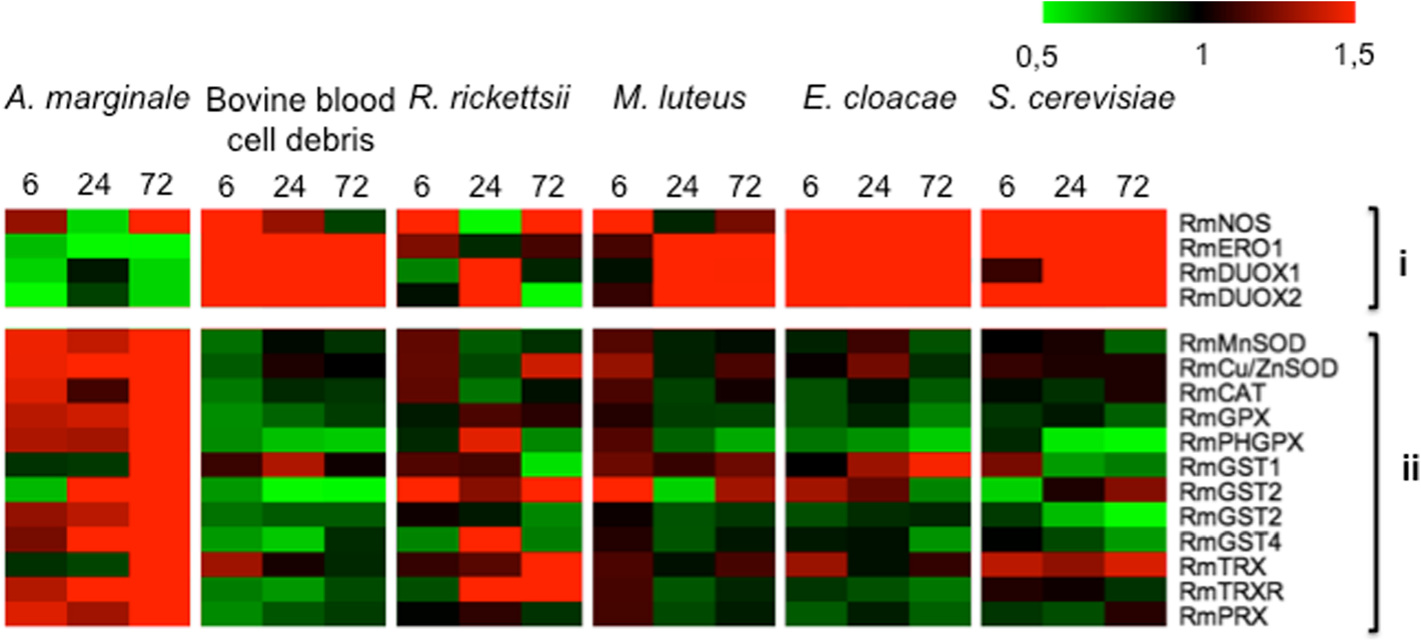
Expression profile of oxidative stress genes in BME26 cells in response to microbial stimuli. BME26 cells were infected with either *A. marginale* or *R. rickettsii* or exposed to heat-killed *M. luteus*, *E. cloacae*, and *S. cerevisiae* from 6 to 72 h. The gene expression profile of BME26 cells was determined using high-throughput microfluidic RT-qPCR. The relative expression of challenged BME26 cells in relation to control cells was calculated according to the method 2^-ΔΔCq^. Each cell in the matrix corresponds to the expression level of one gene in a sample (mean values of three biological replicates). The intensity of the colour from green to red indicates the magnitude of differential expression, based on the colour scale at the top of the figure. *Abbreviations*: (**i**) Pro-oxidant genes: endoplasmic reticulum oxidoreductin (*Rmero*1), nitric oxide synthase (*Rmnos*), dual oxidase-A (*Rmduox-A*), and dual oxidase-B (*Rmduox-B*); (**ii**) Antioxidant genes: manganese superoxide dismutase (*RmMnsod*), Cu-Zn superoxide dismutase (*RmCu/Znsod*), catalase (*Rmcat*), glutathione peroxidase (*Rmgpx*), phospholipid hydroperoxide glutathione peroxidase (*Rmphgpx*), glutathione S-transferase (*Rmgst*1-4), thioredoxin (*Rmtrx*), thioredoxin reductase (*Rmtrxr*), and peroxiredoxin (*Rmprx*)

The transcriptional analysis of BME26 cells was evaluated at 6, 24 and 72 h after experimental infection with either *A. marginale* or *R. rickettsii* or stimulation with heat-killed microorganisms (*M. luteus*, *E. cloacae* or *S. cerevisiae*). No microbial growth was observed after plating the heat-killed microorganisms in an appropriate medium. The results revealed a remarkable difference in the expression of redox metabolism genes in BME26 cells experimentally infected with *A. marginale* compared with the other microbial stimuli (Fig. 1). During *A. marginale* infection, most of the pro-oxidant genes were downregulated, except for *RmNOS*, which was upregulated at 6 and 72 h post-infection. However, most antioxidant genes were upregulated along the infection. It is known that redox equilibrium is modulated in the gut of *Aedes aegypti* after a blood meal [30]. For this reason, we analyzed the relative gene expression in BME26 cells incubated with blood cell debris versus PBS (Fig. 1). The upregulation of the vast majority of pro-oxidant genes and downregulation or non-modulation of most antioxidant genes was observed along the 72 h of stimulation. It is of note that this profile is opposite to that observed in BME26 cells infected with the inoculum of *A. marginale* harboured in blood cells. The global analysis of gene expression of BME26 cells in response to *R. rickettsii* challenge showed that most antioxidant genes were downregulated along infection compared with *A. marginale*, while all pro-oxidant genes were upregulated in at least one point post-infection (Fig. 1). After 72 h, all evaluated antioxidant genes were upregulated by *A. marginale* infection, whereas some of these genes were down-regulated by *R. rickettsii.*

The results of the stimulus of BME26 cells with heat-killed microorganisms (*M. luteus*, *E. cloacae* and *S. cerevisiae*) showed that the expression of most pro-oxidant genes was upregulated and the expression of many antioxidant genes was down-regulated. Although the expression pattern of BME26 cells in response to the heat-killed microorganism is similar, this profile is different from that observed for infection with alive *A. marginale* (Fig. 1).

### *Anaplasma marginale* infection is limited by the simultaneous silencing of *Rmcat*, *Rmgpx*, *Rmtrxr* or by *Rmoxr*1 in BME26 cells

**Fig. 2:**
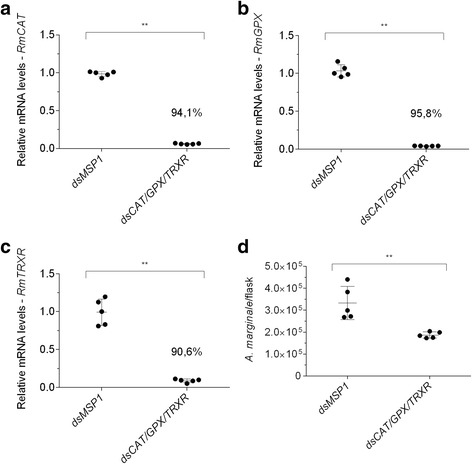
Effect of RNAi-mediated silencing of *Rmcat*, *Rmgpx* and *Rmtrxr* in the control of infection of BME26 cells by *Anaplasma marginale*. BME26 cells were incubated simultaneously with *Rmdscat*, *Rmdsgpx* and *Rmdstrxr*, or with *Pfdsmsp*1 as a control for 24 h and then infected with *A. marginale* for 72 h. The gene expression of BME26 cells was determined using RT-qPCR, and the number of *A. marginale* was determined using qPCR. Relative quantitation of the mRNA levels of *Rmcat* (**a**), *Rmgpx* (**b**) and *Rmtrxr* (**c**) in BME26 cells after incubation with dsRNAs compared with cells incubated with *msp*1 dsRNA (control). **d** Some *A. marginale* cells per flask at 72 h post-incubation in dsRNA-treated BME26 cells. The dots represent the relative mRNA levels or the number of *A. marginale* in individual flasks (five biological replicates), the horizontal line indicates the median relative mRNA level or the number of *A. marginale* cells, and bars represent the standard deviation at each time point. The percentage of gene silencing is indicated in **a**, **b** and **c**. The asterisk indicates significant differences by Mann-Whitney test (*U*_(10)_ = 0, *Z* = -2.88, *P* = 0.0079)

**Fig. 3:**
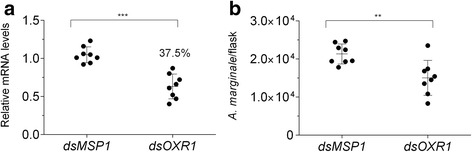
Effect of RNAi-mediated silencing of *Rmoxr*1 in the control of infection of BME26 cells by *Anaplasma marginale*. BME26 cells were incubated with *Rmds oxr*1 or with *Pfdsmsp*1 as the control for 24 h and then infected with *A. marginale* for 72 h. The gene expression of BME26 cells was determined using RT-qPCR, and the number of *A. marginale* was determined using qPCR. **a** Relative quantitation of the mRNA levels of *Rmoxr*1 in BME26 cells after incubation with dsRNAs compared with cells incubated with *msp*1 dsRNA (control). **b** *A. marginale* cells per flask at 72 h post-incubation in dsRNA-treated BME26 cells. The dots represent the relative mRNA levels or the number of *A. marginale* in individual flasks (eight biological replicates), the horizontal line indicates the median relative mRNA level or the number of *A. marginale* cells, and bars represent the standard deviation. The percentage of gene silencing is indicated in A. The asterisk indicates significant differences by Mann-Whitney test (**a** *U*_(14)_ = 0, *Z* = -3.36, *P* = 0.0002 and **b** *U*_(14)_ = 6, *Z* = -2.73, *P* = 0.0047)

As *A. marginale* triggered an antioxidant profile in BME26 cells, the effect of the simultaneous silencing of three antioxidant genes in tick cells, *Rmcat*, *Rmgpx* and *Rmtrxr*, on infection was determined. For this purpose, BME26 cells were incubated at the same time with specific *dscat*, *dsgpx*, and *dstrxr* and then infected with *A. marginale*. The mRNA levels of *Rmcat*, *Rmgpx*, and *Rmtrxr* were 94.1%, 95.8% and 90.6% lower than the control group (*dsmsp*1) (Fig. 2a-c). In addition, a significant decrease in the number of bacteria was observed (Fig. 2d). Likewise, the *Rmoxr*1 silencing (37.5%) (Fig. 3a) resulted in a significant reduction in infection levels of *A. marginale* (Fig. 3b). These results showed that silencing of genes involved with antioxidant defence affects infection of BME26 cells by *A. marginale*.

### Quantification of H_2_O_2_ in BME26 cells infected with *A. marginale*

**Fig. 4:**
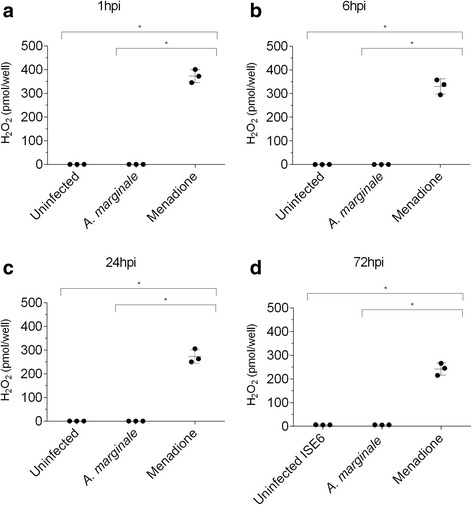
Effect of *Anaplasma marginale* infection on H_2_O_2_ production by BME26 cells. BME26 cells were incubated with *A. marginale*, menadione, or with uninfected tick cell lysate as control (uninfected). The H_2_O_2_ level in the supernatant was quantified using the Amplex Red assay at 1 h (**a**), 6 h (**b**), 24 h (**c**) and 72 h (**d**) post-incubation. The dots represent the concentration of H_2_O_2_ in individual wells (three biological replicates), the horizontal line indicates the median concentration of H_2_O_2_, and bars represent the standard deviation at each time point. The asterisk indicates significant differences by Mann-Whitney test (*U*_(4)_ = 0, *Z* = -2.09, *P* = 0.037)

Since genes involved with redox metabolism are modulated by *A. marginale* infection (Fig 1b), the quantity of H_2_O_2_ was evaluated in BME26 infected at 1, 6, 24 and 72 h (Fig. 4). There was no difference in the concentration of hydrogen peroxide produced by infected and non-infected cells at any experimental time points (Fig. 4). However, menadione stimulation, a known inducer of hydrogen peroxide production [28, 29], increased the production of H_2_O_2_, and the H_2_O_2_ levels varied between 250 and 350 pmol in the studied time points.

## Discussion

In many arthropod species, microbial recognition results in the activation of immune signalling pathways involved in the regulation of important immune-effectors, such as antimicrobial peptides [31]. Indeed, a previous study from our group using heat-killed microorganisms (*M. luteus, E. cloacae* and *S. cerevisiae*) showed that those microbial as stimuli elicited the modulation of many immune-related genes in BME26 cells [17]. Therefore, one of the aims of our study was to verify if those microorganisms could also modulate the expression of redox-related genes. In addition, to evaluate the expression of *R. microplus* redox-related genes during host-pathogen interactions, we have chosen two bacterial pathogens naturally transmitted by ticks, *A. marginale* and *R. rickettsii*. High-throughput qPCR analysis demonstrated that distinct transcriptional patterns of redox metabolism genes are triggered in the cattle tick cell line BME26 in response to different microbial stimuli. *Anaplasms marginale* infection downregulated the expression of pro-oxidants genes and upregulated antioxidant genes. On the other hand, heat-killed microorganisms upregulated most of the pro-oxidant genes and downregulated pro-oxidant genes. Moreover, the gene expression profile induced by infection with *R. rickettsii*, which is not transmitted by the cattle tick, was an intermediary between *A. marginale* infection and the stimulation with heat-killed microorganisms. These results suggest that *A. marginale* infection subverted the host redox-based innate immune response by preventing the onset of an oxidative response, as elicited by the other microbial stimuli.

In accordance with these findings, in our previous study, we observed that *A. marginale* downregulates key components of the signalling immune pathways Toll, IMD, and Jak/Stat, which may reflect an attempt of this bacterium to manipulate the immune system of the host cells [17]. Despite the possible establishment of a favourable environment for the growth of *A. marginale*, we observed a relatively constant number of *A. marginale* over the 72 h infection period. The importance of the maintenance of an antioxidant environment by *A. marginale* was corroborated by either the simultaneous silencing of three antioxidant genes (*Rmcat*, *Rmgpx* and *Rmtrxr*) or *Rmoxr*1 in tick cells, which led to a reduction in the number of bacteria. In turn, even though *R. rickettsii* induces an increase in the expression of most genes of the immune signaling pathways Toll, IMD, and Jak-Stat after 24 h of infection [17] and possibly creates a more pro-oxidant environment compared with *A. marginale* (*RmDUOX-A* and *RmDUOX-B* were induced at 24 h post-infection), we observed high rates of *R. rickettsii* growth and mortality of BME26 cells. A previous study showed that antioxidant genes of *R. rickettsii* are upregulated in the midgut of its tick vector during the acquisition of the blood meal [32]. In addition, antioxidants genes of *R. rickettsii* are also upregulated in endothelial cells exposed to an oxidative environment [33, 34]. Upregulation of antioxidant genes might protect this bacterium against ROS, which may explain its significant growth rate in BME26 cells.

The microbicidal role of ROS produced by professional phagocytes in innate immunity of vertebrates is well known [35, 36]. In addition, it has been established that DUOX is involved in remodelling the intestinal microflora and elimination of unwanted microbes for the maintenance of the gut-microbe homeostasis of the fruit fly *Drosophila melanogaster* [37]. Another study demonstrated the involvement of ROS in mosquito immunity, which is important for the control of both bacteria and *Plasmodium* [13]. However, only a few studies have evaluated the role of ROS in microbial colonization in ticks. To that end, we quantified H_2_O_2_ at 1, 6, 24 and 72 h post-infection of BME2626 with *A. marginale*. It was not detected an augment of H_2_O_2_ production along the course of infection in relation to the control, in contrast to the observed with menadione (positive control). A hypothesis to explain this result is that *A. marginale* infection does not trigger an oxidative response in BME26 cells, which w result in an increase in ROS production, such as H_2_O_2_. In fact, the expression of antioxidant genes was upregulated as early as six h post-infection with *A. marginale*, and their expression increased at 72 h. However, future studies, such as the measure of the activity of antioxidant enzymes, should be performed to corroborate this hypothesis.

To date, few studies have described the involvement of antioxidant defence in the response of ticks to pathogen infection. It was previously demonstrated that the glutathione-S-transferase (*GST*) transcript was induced in IDE8 cells (derived from the tick *I. scapularis*) in response to *A. marginale* infection [38]. Here, a similar transcriptional profile of *Rmgst* genes was observed in BME26 cells infected with *A. marginale*. Importantly, the RNAi-mediated silencing of *gst* in *Dermacentor variabilis* caused a reduction of *A. marginale* load in the tick gut after acquisition feeding and in the salivary glands after transmission feeding [38]. In the current study, we have demonstrated that *Rmcat*, *Rmgpx*, and *Rmtrxr* are upregulated up to 72 h after infection of BME26 cells with *A. marginale*. In addition, we showed that the simultaneous silencing of these three genes decreased the *A. marginale* load, suggesting that they might play an important role in bacterial survival. It has been demonstrated that the viability of *B. burgdorferi* in *I. scapularis* salivary glands are affected by the ROS balance [39]. Moreover, the RNAi-mediated silencing of the peroxiredoxin Salp25D decreased the bacterial load in the salivary glands of ticks, suggesting that this protein facilitated the entry of *B. burgdorferi*. Salp25D is involved in the quenching of extracellular O_2_^-^ produced by neutrophils, allowing bacterial survival [39]. In addition, we have presented evidence here that the silencing of *oxr*1, which regulates expression of enzymes that detoxify ROS, such as catalase and GPX, led to a reduction in the *A. marginale* infection. Similar results were demonstrated for *A. gambiae* infected by *P. berghei.* However, the molecular mechanism of action of *oxr*1 remains unclear [11].

## Conclusions

Herein, we show that different microbial stimuli trigger distinct gene expression profile of components of the pro-oxidant/antioxidant system in BME26 cells. Remarkably, *A. marginale* exposure triggers the downregulation of pro-oxidant genes and the upregulation of antioxidant genes, suggesting that this pathogen might manipulate the oxidative response of BME26 cells to favour bacterial survival towards a more reduced intracellular environment. It is plausible to suppose that the distinct gene profile triggered by *A. marginale* compared with the other microbial challenges evaluated in this study and previous results on immune signalling pathways [17] may be due to the *R. microplus*-*A. marginale* co-evolution. This co-evolutionary scenario may include the adaptation of rickettsial-host interaction to rickettsial advantage, establishing an antioxidant environment and reducing the host immune genes, as well as low rickettsial virulence level, as we observed a low replication rate of *A. marginale* and a high survival of infected BME26 cells. Our results indicate the potential use of BME26 cells to elucidate the molecular mechanisms involved in the infection with *A. marginale*. The mechanisms used by this pathogen to manipulate the immune-triggered production of ROS/RNS are a potential target for the development of new strategies for the control of tick-borne diseases.

## Additional file

Additional file 1: Table S1. Primers used in RT-qPCR analysis and RNAi assays. (DOC 61 kb)

## Abbreviations

CAT: Catalase
DUOX: Dual oxidase
ERO1: Endoplasmic reticulum oxidoreductin
GPX: Glutathione peroxidase
GST: Glutathione S-transferase
H_2_O_2_: Hydrogen peroxide
NO: Nitric oxide
NOS: Nitric oxide synthase
O_2_^-^: Superoxide anion
OXR1: Protein oxidation resistance 1
PHGPX: Phospholipid hydroperoxide glutathione peroxidases
PRX: Peroxiredoxin
RNS: Reactive nitrogen species
ROS: Reactive oxygen species
SOD: Superoxide dismutase
TRX: Thioredoxin
TRXR: Thioredoxin reductase
