# Supplementary material for: Immune-related redox metabolism of embryonic cells of the tick Rhipicephalus microplus (BME26) in response to infection with Anaplasma marginale
Source: Parasit Vectors. 2017 Dec 19;10:613. doi: 10.1186/s13071-017-2575-9 (PMC5738103; doi:10.1186/s13071-017-2575-9)
Supplement: Additional file 1: Table S1. — Primers used in RT-qPCR analysis and RNAi assays. (DOC 61 kb) [file 13071_2017_2575_MOESM1_ESM.doc]

Additional file 1: Table S1. Primers used in RT-qPCR analysis and RNAi assays.

| Gene abbreviations | Gene | GenBank  [number] | Primer-Fw (5’ – 3’) and primer Rv (3’-5’) | Amplicon (pb) |
| --- | --- | --- | --- | --- |
| *RmRIBS3A* | 40S ribosomal protein S3A | [CV443892] | Fw 5' GGACGACCGATGGCTACCT 3'  Rv 5' TGAGTTGATTGGCGCACTTCT 3' | 69 |
| *RmMALATE* | Malate dehydrogenase | [CV455409] | Fw 5' AAACTGTCCCAGTACGAGTC 3'  Rv 5' TTCTTGACGAAGTCCTCTCC 3' | 89 |
| *RmGLUDY* | Glutamate dehydrogenase | [CK185727] | Fw 5' GCCAGACAAATCATGCGTAC 3'  Rv 5' AGCTAAGTGAAGGTGAGACC 3' | 128 |
| *RmCYTORED* | Cytochrome c oxidoreductase | [CK173844] | Fw 5' GGGAAGCTTCTGTTGACATG 3'  Rv 5' ATCAACCACTCTGGGTTCTG 3' | 79 |
| *RmNOS* | Nitric oxide synthase | [CV438673] | Fw 5' CCAATGACAAAGAAGTGCGG 3'  Rv 5' ACTAATCCGCATACGACAGC 3' | 119 |
| *RmERO1* | Endoplasmic reticulum oxidoreduction | [CV453012.1] | Fw 5' ATGGCCCATACACGACATCC 3'  Rv 5' ACGTTGATACTGGCGTGTAG 3' | 97 |
| *RmDUOX1* | Dual oxidase 1 | [CV442800.1] | Fw 5' CGTTACTCAGTGTCTCACTC 3'  Rv 5' ATGACTGCGCAACACATGTG 3' | 141 |
| *RmDUOX2* | Dual oxidase 2 | [CV449552] | Fw 5' CAGAATGTTCGCCTTAGTCG 3'  Rv 5' CGATGTCGTACATGTCGAAC 3' | 134 |
| *RmMnSOD* | Manganese superoxide dismutase | [CV442321] | Fw 5' AGACGCAGAGGCTACAAGTG 3'  Rv 5' AGTTGGCAATGTCCCAGATG 3' | 165 |
| *RmCu/ZnSOD* | Cu-Zn superoxide dismutase | [CK178001] | Fw 5' AAGGTGGGACAAACGATAGC 3'  Rv 5' CGCAAGTAGCTTCAAACACC 3' | 175 |
| *RmCAT* | Catalase | [CK175079] | Fw 5' TTCATGGAGATGTTGACCGC 3'  Rv 5' TGAAGTCCTGAGCATCCTTC 3' | 144 |
| *RmdsCAT* | | | Fw 5´ TAATACGACTCACTATAGGGCCTT  ACCTCTAACATCGCC 3'  Rv 5´TAATACGACTCACTATAGGTCTAG  TAGCGTGATGCAGC 3' | 275 |

| *RmGPX* | Glutathione peroxidase | [CV440147] | Fw 5' TCTCTGTTGGTGACTGAGAC 3'  Rv 5' TGAGTGTTCAGTCCATGGTG 3' | 184 |
| --- | --- | --- | --- | --- |
| *RmdsGPX* | | | Fw 5´ TAATACGACTCACTATAGGCAAGG  ACATTGAGGCCTATG 3'  Rv 5´ TAATACGACTCACTATAGGTTCCAG  AAGGCACATCGTAC 3' | 392 |
| *RmPHGPX* | Phospholipid hydroperoxide glutathione peroxidase | [CK174162] | Fw 5' AAGTATGCAGAGTCCAAGGG 3'  Rv 5' CCTTGCTGAACATGTCGAAC 3' | 136 |
| *RmGST1* | Glutathione-S-transferase | [CK180220] | Fw 5' AGCAGCCAATGGCAACATTA 3'  Rv 5' TTCGCACTCCTTCACCTCAT 3' | 100 |
| *RmGST2* | Glutathione-S-transferase | [CK191620] | Fw 5' ACACTTGTCTTCGAGGTTCC 3'  Rv 5' TAAGCGTACTCAAGCCGGTG 3' | 136 |
| *RmGST3* | Glutathione-S-transferase | [CK191255] | Fw 5' TTACGAAGGGTTCGACACTC 3'  Rv 5' ATACTTGTGGTACTTGTCCGAC 3' | 139 |
| *RmGST4* | Glutathione-S-transferase | [CK178638] | Fw 5' TGAAGGGTGACTACCTCAAG 3'  Rv 5' TGAGGTTGGCGAAGTCCTTC 3' | 177 |
| *RmTRX* | Thioredoxin | [CK190673] | Fw 5' TGGTACGCCCTCAAGTATGG 3'  Rv 5' GTCCATTGGCAGTGATCACG 3' | 121 |
| *RmTRXR* | Thioredoxin reductase | [CV451339] | Fw 5' CAGACTTGGATGCCACCATC 3'  Rv 5' ACACGGAGGGTGTTACTATG 3' | 185 |
| *RmdsTRX* | | | Fw 5´ TAATACGACTCACTATAGGGCGAT  GTCCAGTGTGACTAC 3'  Rv 5´ TAATACGACTCACTATAGGTCACA  GTCAGGGTCGTGAAG 3' | 372 |
| *RmPRX* | Peroxiredoxin | [CK179571] | Fw 5' TGATGACAAGGGCAACCTTC 3'  Rv 5' GTAGGGATGATCGTATCTCC 3' | 162 |
| *PfMSP1* | Major merozoite surface antigen from *Plasmoduim falciparum* | [AF061132.1] | Fw 5´ TGTTGGTTGTCCTCTTTCCCGATG  T 3'  Rv 5´ TTGTCGACTTCATGTTTGGCGGT  G 3’ |  |

| *PfdsMSP1* | Fw 5’ TAATACGACTCACTATAGGCTGAT  GCAAGCGATTCAGAT 3’  Rv 5’ TAATACGACTCACTATAGGGTGTAT  TTCCAGAATTGGCC 3’ | 666 |
| --- | --- | --- |
